# How Do Networks Reflect Collaborative Governance? The Case of a Sport Policy Program

**DOI:** 10.3390/ijerph18147229

**Published:** 2021-07-06

**Authors:** Kati Lehtonen, Petri Uusikylä

**Affiliations:** 1 LIKES Research Centre for Physical Activity and Health, Rautpohjankatu 8, 40700 Jyväskylä, Finland; 2 Social and Health Management, School of Management, University of Vaasa, Wolffintie 34, 65200 Vaasa, Finland; petri.uusikyla@uwasa.fi

**Keywords:** social network analysis, collaborative governance, physical activity, sport policy

## Abstract

This study examines the extent to which collaborative governance thinking is realized in a government-funded sport policy program. Our main argument is that this conversion requires the analysis and interpretation of meso-governance and related networks. In the analysis of meso-level sports policy governance networks, we apply social network analysis and theme interviews. The empirical analysis conducted in this study involves the network-based structure of the Finnish Schools on the Move (FSM) program, which was implemented in Finland from 2009 to 2018. Our research questions are as follows: (1) How have the nature and network structure of the program changed throughout the implementation of the program? (2) How is the collaborative governance thinking reflected in the design and implementation of the FSM program? This study shows that the network is the most significant intermediary structure at the policy and organizational levels. Particularly, it was important in the start-up phase of the program, when the main task of the network was to bring together different actors and to generate a common vision. By the end of the program’s funding, the structure of the network became centralized, with decreasing density. However, the analysis shows that collaborative governance thinking is mainly reflected in implementation, rather than in the genuine joint planning of activities.

## 1. Introduction

The government’s sport policy in Finland has traditionally been pursued through rigid top-down regulatory and budgetary control. However, in the member states of the European Union, program- and project-based thinking has strengthened over the last 20 years, as both EU and national funding have been channeled down to regional and local government through the program structure.

In recent years, alongside program-based policy models, network-like approaches and coordination mechanisms have also emerged. This has been the result of an increase in the interdependence and complexity of the governance system. Self-organized and broadly inclusive approaches to management are a natural response to this increasing complexity [1]. Complexity in this context refers to, among other things, increased interconnectedness and networking, as well as a sudden rise of wicked problems [2,3,4]. The development of the governance paradigm has led to a widely shared understanding that public administration is not able to face this complexity and deal with phenomenon-based policies alone, and there is a need to mobilize a wide range of societal actors [3,4,5]. This is also partly due to the fact that, with the increase in complexity, the ability of public administration to guide society and actors in society has weakened.

However, there seems to be a shared understanding of the basic principles of governance that cuts across trends in different governance. First, governance emphasizes the importance of networks [6,7]. In a complex operating environment, the role of public administration (ministries and municipalities) in practice is to only be one actor among many network actors. It is also often the case that the network is self-organizing, without the need for the involvement of public administration in its creation. Therefore, hierarchical top-down leadership and control lose their meaning. Networking activities also involve cross-cutting cooperation, in line with the “whole-of-government” approach [8].

Governance reforms are a type of perpetual-motion machine used to develop solutions to various problems in public policy. Over time, these will create new public policy problems that require resolutions of a different method [9,10]. The public governance reform policy typically proceeds through contradictory objectives, which are reflected as illogical decision-making regarding governance practices and structures [11]. Bearing such observations in mind, the approach to public governance reform should be consistent and incremental. It is essential to take the traditions of political-administrative culture into account; nevertheless, reform policies should be aimed at the achievement of a long-term vision for the development of governance policies [12].

**Figure 1:**
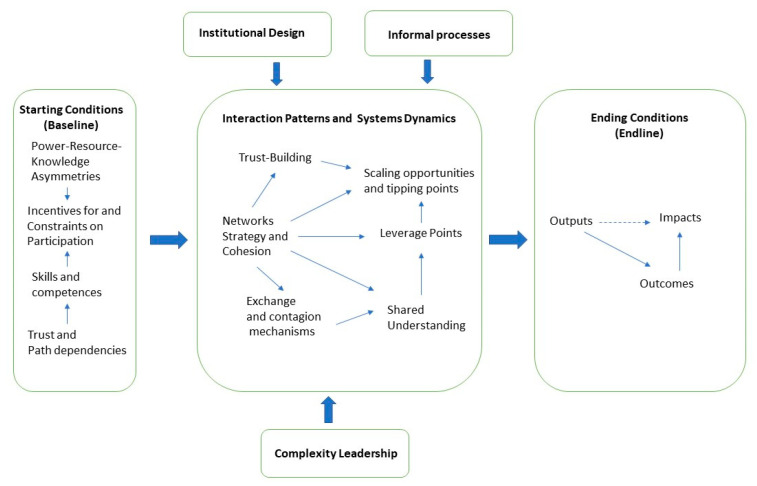
Finnish Schools on the Move as a collaborative governance arrangement.

Collaborative governance (CG) and its practices have been seen as a response to a situation, whereby it is necessary to implement policy actions together with cross-sectoral actors and practices [13]. In this study, we examine the extent to which collaborative governance thinking and network structures are realized in a government-funded sport policy program called Finnish Schools on the Move (FSM), in which the goal is to increase pupils’ level of physical activity in comprehensive schools. Our research questions are as follows: (1) How have the nature and network structure of the program changed throughout the implementation of the program? (2) How is the collaborative governance thinking reflected in the design and implementation of the FSM program? Figure 1 outlines the Schools on the Move Program from a collaborative governance perspective.

The basis for collaborative governance is determined by the starting conditions (i.e., the baseline). Power relations, asymmetries, and epistemic tensions limit opportunities for action and affect incentives and constraints. The skills and abilities of different groups of actors are of great importance for the type of operating conditions under which they need to achieve their goals. The actual collaboration takes place in an interactive systemic space [14]. Networks and operational strategies are at the heart of the analysis. They determine the relationships between actors, sociometric distances, and structural holes [15]. Trust and exchanges are of great importance for the type of transactions that take place in networks. Shared understanding and opportunity creation open up channels of influence, that can have rapid leverage effects on an entire system. Institutional design and informal processes determine which opportunities for networks in the field of systemic activity are available.

Complexity leadership is a key tool for controlling system dynamics. Ultimately, the action will not be of great importance unless it produces results and genuine change in the environment. Complexity leadership theory (CLT) is also acknowledged in the conceptual framework. It is developed in an organizational context, and emphasizes that the adaptive space of leadership is created when informal and formal types of knowledge unite [16]. This is considered as a state of embracing and benefiting from complexity, instead of following the traditional leadership methods to repress it. When brought to a context of leadership, complexity retains its profound dynamic of “both and”. For example, leadership does not consist only of deliberative actions and developments, but also of self-organizing and emergent events [17]. In CLT, this is explained through ambidexterity, which parallels the formal and informal sides of organization and leadership. The key factor in this theory is how we enable connections with (1) a local informal system that explores and deals with situations and (2) a formal operational system that exploits ideas and operationalizes them as a new order for the whole system. Hence, the premise is that the local informal system must innovate, and the formal operational system must produce.

In the field of sport governance research, CG may be an integrative framework to identify relevant questions for new research directions in sport governance [18]. For example, as the field of sport has become more professionalized, and phenomena such as sedentary behavior has become a general governmental issue, old sport structures and customs that are based on traditional actors and governance mechanisms no longer offer sufficient solutions [19].

As a CG research agenda, three relevant research themes have been pointed out: power and structure, leadership and motivation, and decision making [20]. Thematically, researchers have focused on the sport federal model as a type of network [18]; trust as a mechanism to manifest and impact on the level of collaboration that take place in sport governance networks [21]; and collective leadership in governance decision making by analyzing an intervention that is designed and implemented with the Bowls Australia Board [22]. A case study approach in the context of Australian sports was used to examine the contributing factors that facilitate or inhibit trusting relationships between boards within sporting networks. The degree to which trust, transparency, the capacity to build trust, and leadership were embedded in the cultures, as well as the processes of each network, varied significantly. As a key finding, leadership was found to be an important factor in fostering collaborative relations at the different levels of governance [21].

Later, the term “collective board leadership” was used to describe the leadership style that might be best suited to collaborative governance. Board-to-board relationships were researched to generate key strategic leadership and progress initiatives within and across a network of affiliated bodies, such as in a federated model, in which several independent organizations constitute a network-based system [22]. However, it seems that the concept of collective board leadership was unfamiliar, and it did not resonate with the directors of the Australian golf network [23].

When summarizing studies concerning CG in sport, two main conclusions are relevant. First, the previous studies relate to sport more than to physical activity, which means that the role and structure of sport organizations and sport systems are more relevant than in our current study, whereby the focus is on a state-controlled program aiming to increase the physical activity of children and adolescents. As a consequence, the logic of actors in the program’s networks are mainly connected to current sport policy action, focusing on the public sector’s school environment and physical activity. The network they constitute is established due to the need to govern the program, not to sustain a (federal) system. However, these different premises are not in conflict when looking at the possibilities, which have been explored as a research agenda for CG and its theoretical base. Therefore, this study complements the research agenda framed by the CG theory, from a new perspective focusing on a sport policy program based on network practices. To be specific, the analysis of the network of actors in the FSM program provides new insight into collaborative governance. through the application of the network analysis of the structural characteristics of the FSM network.

As for the societal impact of the study, this article presents possibilities for increasing the strategy of the network governance by analyzing the CG practices that the actors have produced in the FSM program. As a result, it provides tools to develop public governance related to sport. Belonging to, acting in, and working in networks necessitates more understanding of these networks as a contributor to public sector effectiveness and forms of governance. There is also a methodological contribution, in combining social network analysis (SNA) and qualitative themed interviews. This solution is rarely used in sport social sciences [24,25,26], despite the research concerning SNA in sport social science is increasing [27].

## 2. Data and Methods

This research study relied on the use of a mixed methods approach. The collection and analysis of the data were based on methodological triangulation, an approach in which more than one method is used to collect and analyze data. This is not only a way to assure the validity of the research, but also a means of capturing multiple dimensions of the phenomenon being researched [28]. In terms of research design, a sequential exploratory design was chosen. This design allows the researcher to first collect quantitative data, and then use qualitative data to explain the mechanisms underlying the results of the quantitative data [29]. The data were collected through SNA and theme interviews with key informants. Thus, in the context of the current study, the role of the theme interview data are to complement the SNA data, to obtain a more productive, realistic, and detailed understanding of the implementation and governance of the FSM program. More specifically, the study aimed to gain more insight into two internal mechanisms, through the collection of the interview data. First, the interview data help in explaining perspectives on the decision making of the state officials, whom are responsible for state funding and sport policy implementation and are representative of the FSM office, which is in charge of coordinating the program. Secondly, interviews provide possibilities to interpret the SNA results and examine the structure of the network-based program and CG practices. However, we first present the case—namely, the FSM program—to create an overall impression of its main focus and progress as a sport policy action.

### 2.1. The Case: The Finnish Schools on the Move Program

In a policy session carried out in 2009, the Finnish government took a stand on promoting good conditions for physical activity among children and adolescents. In their statement, the government defined the actions needed to ensure that the targets of the government program would be met. One of these actions was Finnish Schools on the Move, with its goals emphasizing the need to increase pupils’ physical activity in comprehensive schools. In practice, this meant establishing a day-to-day operating culture involving physical activity in schools, rather than only increasing the quantity of physical education classes [30].

The program was launched as a project, and in spring 2010, during the pilot phase, funding was granted to 21 municipal projects involving 45 schools. The funding that was allocated was nearly 450,000 euros. The project funding for municipalities came from lottery funds, and the municipalities could allocate the funding freely to their chosen school setting, in line with the main project goal. This municipality-specific funding model is still in use today.

In the pilot phase, from 2010 to 2012, the project’s focus was on increasing the overall PA, which in this model meant a blend of sport, physical games, play, and outdoor recreation activities. As the project became a national program in 2013, the content and goals increasingly highlighted other concepts, such as making school days and learning more enjoyable. However, the 2015 government program included an item that was especially significant for the progress of the program: the government’s key project on new learning environments and digital materials for comprehensive schools included a procedure for reaching the target of ‘one hour of physical activity per day’ [31].

By late 2018, 90% of Finland’s comprehensive schools had registered as participants in the FSM program, which corresponds to a total of 2136 schools. Of the more than 300 municipalities in Finland, 93% took part in the program, including more than half a million basic education pupils, representing 92% of pupils at this level [32]. Registration for the program entails the school becoming part of the school network. Financial support for the program also increased, most noticeably between 2016 and 2018, when it amounted to 21 million euros for the programming period. Most significantly, the project funding for municipalities over the past two years has come directly from the state budget.

From its inception, the program has been based on networked co-operation at the administrative level and in stakeholder groups [33]. This was already required in the pilot phase; the appointment decision for the program highlighted multisector cooperation among ministries, organizations, and other stakeholders [32].

### 2.2. Social Network Analysis

A social network is a set of socially relevant nodes, connected by one or more relations. Nodes, or network members, are the units connected by the relations, and it is the patterns among these that are under study. These units are most commonly people or organizations, but any units that can be connected to other units may be studied as nodes [34]. For this article, social network analysis was used to improve the research perspective on the structural changes in the FSM program’s network between 2009 and 2018. To examine the change in network governance over the years, the data set was split into two periods: 2009–2014 and 2015–2018. This periodization was based on the FSM program’s status as a national program: in the latter period, FSM was a part of the government program and had a high status.

The data for SNA were collected from the sport policy memos and the FSM program’s documents, which consisted of archival data. This type of data is more useful than questionnaires, for example, when the aim is to explain structures in certain social networks [35]. The network consisted of governance within the state administration, including all steering, preparation, and management groups; advisory boards; and theme-based sub-groups that had been part of the program (*n* = 14). All of these groups were established and named by the state. The total number of separate organizations represented in the groups was 58. The inclusion criteria were membership in a project, working group, or committee. These memberships have been referred to as events. In network analysis, two-mode data are frequently encountered in the form of affiliation data: information is collected on a set of groups or events that a set of actors are involved with. The data matrix typically has actors for rows, and groups or events for columns. A given data value X(*i*,*j*) = 1 if actor *i* is associated with group/event *j* and X(*i*,*j*) = 0 otherwise. From this matrix, we computed actor-by-actor matrices by counting the number of groups/events that each pair of actors have in common. The result is a “co-occurrence” or “co-membership” matrix that is then analyzed as ordinary (valued) network data. Alternatively, one can compute an event-by-event matrix, that counts the number of actors attending both events for every pair of events, yielding a measure of the overlap of attendance between the events. 

The data analysis was performed with two-mode analysis in the UCINET network analysis software [36]. The names of the organizations represented in the groups were entered in an Excel spreadsheet, after which data analysis for a two-mode network was performed in a matrix format. The data in the cases of two-mode networks involved a set of binary relationships between members of two sets of items [37]. To analyze a social network of this sort, one must usually have data on which actors have participated in which events. In this study, the state’s groups, such as the Steering Group or Advisory Board, were used as events and the organizations who were members of them were the actors. As the actors’ structural properties were analyzed, the focus was on the affiliations, the links constituting the affiliations, and the centrality of the actors. The core idea behind measuring centrality is simple: actors who are more central in social structures are more likely to be influential and powerful; they may extract better bargains and, in the context of this study, have greater opportunities to control the network [37,38]. In this study, the degree of centrality was considered because it best indicates the key actors from the standpoints of coordination and control.

The degree of centrality explicitly describes the number of links that a given actor or event has. In the case of this study, the degree is the number of links to events. Hence, the degree of centrality is used to measure the activity or direct influence of a certain actor [39]. Actors with a high degree of centrality have access to and can call on more of the resources within the whole network. They are also often third parties and dealmakers in, among other things, exchanges, and they can benefit from this brokerage. Therefore, degree is often a simple but effective measure of an actor’s centrality and power potential. Moreover, although centrality measures are the most common ones used in SNA, density is also essential. Density describes the cohesion of the network, and was calculated in this article by dividing the total number of actual ties by the total number of possible ties [38].

### 2.3. Interview Data

Thematic interviews were conducted with government officials that were representing the Division for Sport of the Ministry of Education and Culture (*n* = 4), and the Finnish Schools on the Move Office (*n* = 1) between October and December 2018. The interviewees were key informants on the issues related to the governance model of the FSM program. As stated, a key informant is an expert source of information [40]. The key informants—i.e., the interviewees—were selected because of their formal role and position, as well as their knowledge related to the program´s administration. All interviewees participated voluntarily on an informed consent basis, with the possibility to withdraw at any time, and with guaranteed anonymity. Due to the latter ethical criteria, detailed working titles and affiliations are excluded.

In addition, while the number of interviewed people is small, text examples are given in the results section, using standard language to guarantee the anonymity of informants. In addition, the interviewed people were not coded, but rather separated based on their background organization of the Ministry of Education and Culture (MEC) or the Finnish Schools on the Move Office (FSM office).

The same thematic questionnaire was sent to every interviewee before the interview by e-mail. All interviews were recorded, conducted, and analyzed in Finnish, as the natural language of the informants and researchers. The selected text examples were translated into English when writing the results section. The total duration of the interviews conducted was 5 hours, which produced 22 pages of transcribed text (11-point Calibri text with single line spacing). The average duration of an interview was 60 min, with a range from 50 to 77 min. The interviews were conducted in the offices of the informants. Two broad areas provided the thematic structure for the interview framework: (1) the network-based governance model within the state administration and (2) the design and implementation of the program. As an example, the detailed questions were: How is the network-based FSM program organized in the Ministry of Education and Culture? In what terms could network leadership be developed in the FSM program?

The material was analyzed in three phases by one researcher using theory-based content analysis [41]. In the first reading, pieces of material were classified into the aforementioned general thematic areas. After that, the data were classified based on collaborative governance thinking, from the viewpoint of the design (baseline) and implementation of the program (interaction patterns and systems dynamics), as presented in Figure 1. While doing so, the periodization of SNA was followed, making it possible to analyze how the interviewed people explained the evolution of the FSM program. In the third phase, the framework of CG was deepened, and attention was paid to particular matters, such as what the interviewees said about the program’s governance practices in the name of path dependency or trust. Finally, the text corpus was divided by thematic area into the following topics: power resource practices, trust, path-dependency, shared understanding, strategy, institutional design, complexity leadership, and network-based governance. In the results section, each coded topic is presented along with examples from the text.

## 3. Results

### 3.1. Quantitative Analysis; SNA

In both periods (2009–2014 and 2015–2018), public sector organizations were found to have the most affiliations with the FSM program’s network. In the first period, the share of affiliations was 55% and in the second period it was 58.3%. The share of third sector organizations’ affiliations decreased between the first period (37.7%) and the second (32.6%). The share of the FSM office’s affiliations increased slightly, from 7.3% to 8.7%. In the second period, one organization from the private sector obtained one affiliation with the thematic sub-group (0.5%).

The number of events increased from 3 to 12, and the number of actors increased from 22 to 38 (Table 1). In addition, the number of affiliations per period increased from 43 to 103. This means that the network expanded, and the structure gave actors more opportunities (events) to influence. However, the density of the network decreased from 0.652 to 0.226, which meant that the cohesion of the network decreased.

In both periods, the minimum number of affiliations per actor was one. The maximum number of affiliations per actor increased from three to nine. In addition, the share of affiliations of those actors who had three or more affiliations increased to 76% in the second period. The share of actors in this group also increased from 32% to 42%.

At the same time, the share of actors with the maximum degree of centrality decreased from 31.8% to 2.6%. In the second period, there was only one actor, the FSM office, with the highest degree of centrality (0.750), which was the most central actor in the network structure. Overall, the structure of the network was more centralized in the second period than it was in the first.

**Figure 2:**
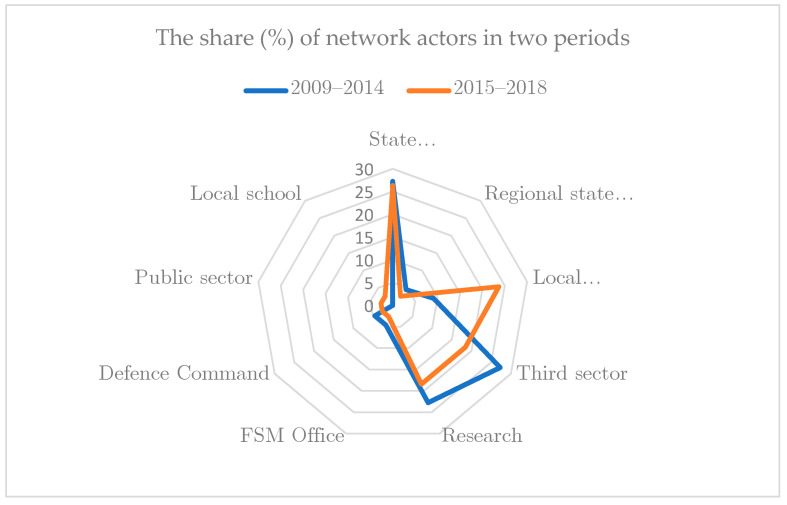
The share (%) of network actors in the two periods.

However, when classifying actors based on societal sectors and administrative positions while only considering the number of actors between the periods, the share of municipalities as local administrative actors increased remarkably (Figure 2). In the first period, the share of local administration representatives was 9%, and in the second period it was 23.7%. Comparably, the share of third-sector actors decreased (27.7% to 18.4%), as did the share of research organizations (22.7% to 18.4%).

**Figure 3:**
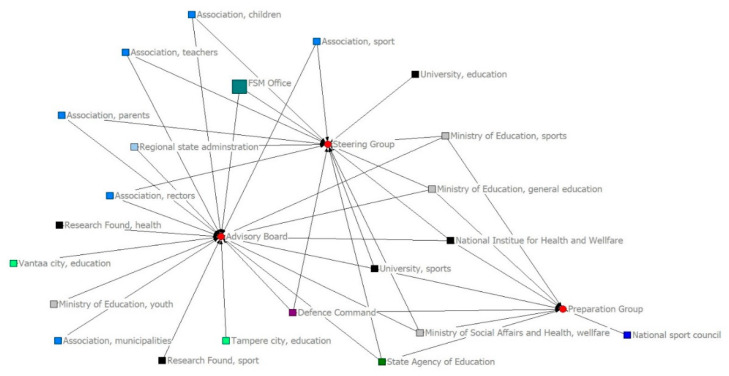
FSM program’s governance network in the first period (2009–2014).

**Figure 4:**
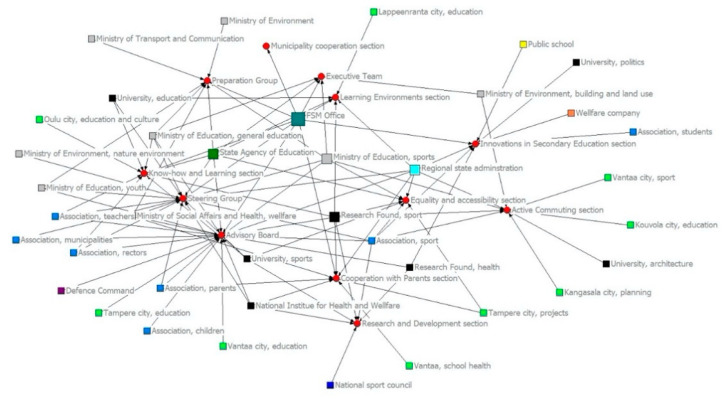
FSM program’s governance network in the second period (2015–2018).

Figure 3 and Figure 4 illustrate the structural changes between the two periods. The FSM office is highlighted in a different color and size because of its role in the program. Overall, the different colors match different actor types with one another, such as association, ministries, and municipalities, in order to obtain an overview of the structure. The role of the FSM office was not central in the first period, but in the second it was the core of the network. Along with the office, the coordinating nexus of the network consisted of the Ministry of Education and Culture (Division for Sport), the State Agency of Education, the Regional State Agencies, and the Research Foundation (sports). As in the first period, the positions of actors in different events were two-fold. The main management groups of the program were on the left side, where actors with affiliations with these events mainly came from state administration or other public organizations and school-related associations.

The share of municipalities as local administration representatives increased in the second period, and the aim of the program was local level school-based operations. Nonetheless, only two municipalities had representatives in the program’s management group (advisory board). However, the main affiliations of the municipalities were linked to events that were focused on the thematic planning of the program, such as equality, accessibility, and learning environments. It seems that the role of local actors was practical, not managerial, from the viewpoint of the program’s management practices at the national level.

### 3.2. Qualitative Analysis: Interviews

The interviews revealed that the collaborative governance arrangements and thinking occurred in relation to the following themes: path dependencies and power resource (a)symmetries (baseline/starting conditions), strategy/strategic planning, and trust and shared understanding (interaction patterns and systems dynamics). In addition, forms of complexity leadership and institutional design were found (Table 2).

When the FSM program was in its planning phase (baseline), the solution for establishing the program and the FSM office as part of the National Agency for Education was self-evident, while the focus of the FSM program was the school environment. Therefore, the way that the state used its structure and power resource practices while building the program’s governance model was not asymmetric but symmetric.

In addition, the chosen model could be seen as part of the reforms of the sport system that began at the same time as the FSM´s pilot phase, in the early 2010s [42,43]. Through the reform process, the state provided itself with a new target and the possibility of acting as a developer of sports culture and, in particular, physical culture [33]. The program and its governance model were used to break the path dependency of implementing Finnish sport policy, and to show the central sports organizations their new ‘place’ that had room for action within the networked system.

From the viewpoint of implementation (interaction patterns and the dynamics of the system), the main goal was to gain a shared understanding of the creation of a new network-based governance model coalescing around the FSM program. In addition, an important element was to have a vision of the coordination and program planning and what it entails—i.e., strategic planning was carried out. However, the basis of the implementation and networking was trust, which was built together with commitment, shared understanding, and strategic practices. While the FSM office acted as a coordinator of the program, trust building was mainly in the hands of the office and its actors. Thus, the office can be said to be a trust builder, which was working strategically to find network brokers to enable the program.

## 4. Discussion

The empirical analysis of the FSM program shows that collaborative governance thinking mainly occurs in the way that institutional design determines opportunities for the FSM program´s network, at the systemic level. In the FSM program, the chosen governance model inside the state administration gives credibility to the FSM office and legitimizes its position inside the program´s network. Accordingly, it seems that the FSM office’s external positioning and location influenced not only the legitimacy of the FSM office but also that of said office’s employees for the coordination of networks. These findings strengthen our earlier remarks underlining the importance of legitimacy as a key element of successful networking [44].

In a broader sense, the location of the FSM office was part of this implementation of multilevel governance—also called complexity leadership—which is the element at the core of collaborative governance for controlling systemic dynamics. However, as a single actor, the FSM office could not function as the coordinator of the program. It requires other organs and levels of governance to achieve its full capacity as a coordinator [45]. In the FSM program, complexity leadership followed in two ways. First, multi-sector steering and management groups were set up, featuring representatives from the various ministries. This brought greater political legitimacy to the program, while also strengthening the institutional design of the program [14]. In addition, more seemingly peripheral ministries became further engaged with the program. A practical effect of this was that increasing physical activity throughout the school day was administratively understood as a multi-sector challenge.

Second, the role of the MEC’s Division for Sport was important in coordinating the many differently oriented ministries. However, the division of roles between the Division for Sport and the FSM office was at the heart of the governance model. At the same time, this division opened a door to the operational logic of traditional public administration, in which hierarchies form the foundation for policy actions by using these hierarchies and bureaucracy as the major building blocks. This combination was indeed seen in the governance model of the FSM program, where the coordination of the program included the role of a trust builder and informal relationships handled by the FSM office, while MEC´s Division for Sport were in charge of the funding and formal leadership of the program. Our findings strengthen earlier research findings, in which trust was found to be an important feature of successful collaborating practice in sport-related networks [21,43,46].

The SNA showed that despite the extension of the network in the second period, the structure of the network became centralized, and formal power resources were increasingly placed in the hands of the MEC and FSM office. The latter was important when the aim was to enable someone to coordinate the program. However, from the viewpoint of the implementation and idea of CG, actors from different sectors (private and third) or representatives of local-level administrators were missing when we observed those actors in the nexus of the network. In other words, the state kept the power resources in its own hands, and the complexity leadership was extended mainly to other ministries and public authorities rather than to the executors of the program, i.e., municipalities, which played the role of innovators in different sub-groups. Therefore, we can conclude that the CG practices reflected the state administration well, including the FSM office, but the complexity of the leadership arrangements did not actualize in the wider sense, where self-organizing and emergent events were relevant [17].

Every study has its limitations, and in this current one we would like to point out the scientific generalization of the results, and the utilized mixed method analysis. As we considered the FSM program as a case, it is obvious that the interpretations based on data cannot be widely generalized without some re-contextualization [47]. For example, both the educational and sport system, as well as sport policy guidelines must be taken account if the aim is to repeat this current research design in another country [43]. However, this study on the FSM program was not treated a case study to test the collaborative governance model. Rather, the model was used as a reference framework in order to understand the role and importance of participatory networks in implementing a government sports program. Thus, the limitations, such as the case study selection criteria can be argued to be not applicable in the context of the current study. One aim of this article was to bring together thematic interviews by key informants and social network analysis, as a form of methodological triangulation. The body of interview data was small, but consisted of key informants and their views. It is evident that they do not represent all viewpoints of the FSM program. This solution did somewhat consign other potential informants—such as those involved in the National Agency for Education or with LIKES—to the shadows. However, despite this shortcoming, the data and methods selected proved able to answer our research questions pertaining to the FSM program’s collaborative governance practices. Using SNA as an independent method would not have been sufficiently informative, particularly with regard to the aim of finding explanations for the design of the governance of the FSM. In addition, our focus was on the state-led program, where the Ministry of Education and Culture and FSM office have evident roles as implementors of the program. Based on this viewpoint, the number and selection criteria of key informants were in line with the requirements of the research framework and to ensure validity [40]. Regarding the reliability of the methodological choices, the SNA data were open access archival data, consisting of memos, policy documents, etc. Therefore, the process of collecting this same data and analyzing them using UCINET is possible to repeat.

A key shortcoming of this study is the lack of reliable outcome and effectiveness data. In this study, networks or collaborative arrangements could not be used to explain the results and impacts of the program. This would require a completely different research design—for example, applying a randomized controlled trial design (RCT) for a comparison of the results and impacts of physical activity programs in purely top-down cases and those of the participatory models. Thus, this is recommended as a topic for further research, particularly since systematic monitoring and evaluation data were collected from the FSM program.

## 5. Conclusions and Implications

This study shows that networks are the most significant intermediary structures at the policy and organizational levels. They have been particularly important in the start-up phase of programs, when the main task of the network has been to bring together different actors, and to generate a common vision. However, the analysis shows that collaborative governance thinking is mainly reflected in the implementation, rather than the genuine co-creation of activities. This is related to the instrumental nature and the strong concerns of cooperation and network-based activities, which primarily aim to obtain funding rather than to achieve the consolidation and dissemination of results.

As a practical policy recommendation, it could be suggested that sport policy programs should be prepared through joint planning, co-design, and generating wider involvement. In this case, regional and local executive structures and networks would be likely to survive longer after the end of funding. The current program and funding model encourages sub-optimization and the instrumental development of operations. Solving complex social, environmental, economic, and political challenges is a collective endeavor. These challenges are often multi-dimensional and decentralized. Often, they emerge or change very rapidly, particularly when the environment is unpredictable. In an environment such as this, a sustainable impact on physical activity programs can be achieved only by social innovations that are based on local needs and designed by local actors for the local users.

Finally, when observing the results from a practical perspective, more effort should be devoted to thinking strategically about the particular types of models used for network coordination before one starts to implement the instruments directed at the network-based policy goals. This research has indicated that this is possible. However, a shared understanding of central actors is needed, in order to carry out the strategic work as well as implement it into daily practice.

## Figures and Tables

**Table 1:** Network attributes.

Network Attributes	2009–2014	2015–2018
Number of events	3	12
Number of actors	22	38
Number of affiliations	43	103
Affiliations min per one actor	1	1
Affiliations max per one actor	3	9
Share of actors with 1–2 affiliations	68%	58%
Share of actors with 3 or more affiliations	32%	42%
Share of affiliations of those actors with 3 or more affiliations	49%	76%
Degree centrality max	1.000	0.750
Degree centrality min	0.333	0.083
Share of actors with maximum degree centrality (%)	31.8	2.6
Density	0.652	0.226

**Table 2:** Themes identified in the interviews, with selected text examples (adapted from Finnish to English) for illustration.

Theme	Text Examples
Power resource practices	There was a government program with the goal of establishing the FSM or a pilot scheme and the ministry started to plan it. It was clear that it had to be a combination of a bottom-up perspective at the local level and the state’s steering mechanism (MEC).
Path dependency	From the beginning, it was clear that there must be an external office. For example, the central sport organizations could not have handled program (MEC).
Strategy	There should be a planned nexus in the network which coordinates or controls the node where the ropes go through (MEC).
Shared understanding	There is a need to have more networking when speaking about our sport policy and its implementation. This gives us the possibility to reach a common view (MEC).
Network-based governance	The network-based idea of working is evident in our sport, but perhaps needs refreshing. For example, maybe the actors in networks should change when the times change (MEC).
Trust	If there is no trust, there is no action in the network. Trust building must be strategic. However, it takes time to find the right people. Those who are in the right place in the network can impact the program (FSM office).
Institutional design	Being within the National Agency for Education has been important for the program. It has given the program a structure in which to operate (ME).
	The state gives us the shoulders. It says that the office is the responsible party and that this is the mandate for working as a coordinator. Additionally, schools think that the program is more credible when our office is positioned within the National Agency for Education (FSM office).
Complexity leadership	The Ministry of Education and Culture has opportunities to connect with other ministries involved in this program and utilize their expertise. For example, the role of the Ministry of Transportation and Communication is very important when we are speaking about biking routes and whether children can go to school on foot or by bicycle (MEC).
	The FSM office is in charge of operative managing and coordination. MEC and the Division for Sport are in charge of funding. Additionally, in the endgame, MEC is in charge of the program if something does not stay in line (MEC).
	If something problematic comes up in the program, the management tools must be changed and used—for example, the hierarchy, such as the Division for Sport and its effort. This means that the office must have a good relationship with the Division for Sport (FSM office).

## Data Availability

No new data were created or analyzed in this study. Data sharing is not applicable to this article.
